# Of Cattle, Sand Flies and Men: A Systematic Review of Risk Factor Analyses for South Asian Visceral Leishmaniasis and Implications for Elimination

**DOI:** 10.1371/journal.pntd.0000599

**Published:** 2010-02-09

**Authors:** Caryn Bern, Orin Courtenay, Jorge Alvar

**Affiliations:** 1 Division of Parasitic Diseases, Centers for Disease Control and Prevention, Atlanta, Georgia, United States of America; 2 Department of Biological Sciences, University of Warwick, Coventry, United Kingdom; 3 Department for the Control of Neglected Tropical Diseases (HTM/NTD/IDM), Leishmaniasis Control Program, World Health Organization, Geneva, Switzerland; Institute of Tropical Medicine, Belgium

## Abstract

**Background:**

Studies performed over the past decade have identified fairly consistent epidemiological patterns of risk factors for visceral leishmaniasis (VL) in the Indian subcontinent.

**Methods and Principal Findings:**

To inform the current regional VL elimination effort and identify key gaps in knowledge, we performed a systematic review of the literature, with a special emphasis on data regarding the role of cattle because primary risk factor studies have yielded apparently contradictory results. Because humans form the sole infection reservoir, clustering of kala-azar cases is a prominent epidemiological feature, both at the household level and on a larger scale. Subclinical infection also tends to show clustering around kala-azar cases. Within villages, areas become saturated over a period of several years; kala-azar incidence then decreases while neighboring areas see increases. More recently, post kala-azar dermal leishmaniasis (PKDL) cases have followed kala-azar peaks. Mud walls, palpable dampness in houses, and peri-domestic vegetation may increase infection risk through enhanced density and prolonged survival of the sand fly vector. Bed net use, sleeping on a cot and indoor residual spraying are generally associated with decreased risk. Poor micronutrient status increases the risk of progression to kala-azar. The presence of cattle is associated with increased risk in some studies and decreased risk in others, reflecting the complexity of the effect of bovines on sand fly abundance, aggregation, feeding behavior and leishmanial infection rates. Poverty is an overarching theme, interacting with individual risk factors on multiple levels.

**Conclusions:**

Carefully designed demonstration projects, taking into account the complex web of interconnected risk factors, are needed to provide direct proof of principle for elimination and to identify the most effective maintenance activities to prevent a rapid resurgence when interventions are scaled back. More effective, short-course treatment regimens for PKDL are urgently needed to enable the elimination initiative to succeed.

## Author Summary

Visceral leishmaniasis (VL) is a potentially deadly parasitic disease that affects 200,000 to 300,000 people per year in the Indian subcontinent, where an effort is currently underway to eliminate the disease. Studies have identified fairly consistent patterns of risk factors for VL. This information can help to inform the elimination effort. Because humans form the sole source of infection, clustering of VL cases is a prominent feature, both at the household level and on a larger scale. Mud walls, dampness in houses, and peri-domestic vegetation increase infection risk by promoting the survival of the insect vector. Bed nets, sleeping on a cot and indoor residual spraying decrease risk. The presence of cattle is associated with increased risk in some studies and decreased risk in others, reflecting a complex effect on vector abundance and vector infection rates. Poverty is a major underlying factor increasing risk of VL infection and disease. A well-designed demonstration project is needed to provide direct proof of principle for elimination and to identify the most effective maintenance activities to prevent a rapid return of disease when interventions are scaled back.

## Introduction

South Asia contains the largest visceral leishmaniasis (VL) focus in the world, with an estimated annual incidence of 200,000–300,000 clinical cases [1],[2]. In this region, the disease is caused by the protozoan parasite *Leishmania donovani* and transmitted by the sand fly *Phlebotomus argentipes*; infected humans constitute the only demonstrated reservoir [3],[4]. Infected individuals may develop the most severe clinical syndrome, referred to as kala-azar (“black fever”) and characterized by prolonged fever, wasting, splenomegaly, hepatomegaly, secondary hemorrhagic and infectious complications, and more than 90% case-fatality in the absence of treatment [3],[5]. However, most VL infections are asymptomatic and detected only by serological testing or a positive delayed-type hypersensitivity (DTH) response to the leishmanin skin test [6]–[8]. In many villages with sustained transmission, more than 30% of residents have positive leishmanin skin test results as a consequence of cumulative past exposure; the prevalence typically rises with age [9]–[11]. The cell-mediated immune response indicated by a positive skin test affords strong protection against subsequent clinical kala-azar [9]. In contrast, a positive result by serological testing is thought to reflect more recent exposure, and usually reverts to negative within a year in asymptomatic infections [7]. In the only published comparative incidence study in South Asia, the ratio of asymptomatic seroconversions to kala-azar cases was 4∶1, similar to the 6.5∶1 ratio observed in a cohort of Brazilian children [6],[7].

An estimated 5–10% of clinically recovered patients develop a macular, papular and/or nodular rash known as post kala-azar dermal leishmaniasis (PKDL) within 5 years of kala-azar treatment [12]. A small fraction of PKDL cases appear without prior history of kala-azar [12]. Patients with PKDL are usually asymptomatic aside from the rash, but are infectious to sand flies and are thought to provide a durable infection reservoir, because the condition can last for years or even decades [13],[14]. However, longitudinal studies on the infectiousness of PKDL and kala-azar patients to sand flies that would provide conclusive data on their relative epidemiological importance as reservoirs have yet to be conducted [4].

In 2005, the governments of Bangladesh, India, and Nepal launched an ambitious initiative to eliminate VL in South Asia by 2015 [15]. The regional initiative assumes feasibility of elimination [15] based on the reported disappearance of kala-azar that occurred as a collateral benefit of indoor residual spraying (IRS) with DDT under the auspices of the Global Malaria Eradication Program in the 1950s and 1960s [13],[16]. However, the explosive resurgence of VL, beginning around 1970 in India and continuing in all three countries, highlights the difficulty facing the elimination program [16]–[20]. The objectives of this article were to (1) review and synthesize existing data on risk factors for VL in South Asia, and (2) use this review to inform the epidemiological context of the current regional VL elimination strategy and identify key gaps in current knowledge. We were particularly interested in reviewing epidemiological and entomological data relevant to the role of cattle in mediating human VL risk, because primary risk factor studies have yielded apparently contradictory results.

## Methods

To identify risk factor data, we reviewed the literature based on MEDLINE searches using the term *visceral leishmaniasis* with the subheading *risk factors*, and the geographic terms *India* or *Bangladesh* or *Nepal*. Articles published from 1966 through July 22, 2009 in English were included. The search for risk factor studies yielded 26 potentially relevant articles, of which 10 were retained based on our selection criteria. We included all studies that explicitly addressed factors associated with altered risk of VL in South Asia in which the objectives, design, outcome measures and analyses were clearly described and judged to be adequate and free of avoidable biases based on the methods and data presented. A formal meta-analysis was not feasible because of the sparse data and non-comparability of study designs and outcomes. In this review, measurement of risk is presented as odds ratios plus p values or 95% confidence intervals, extracted directly from tables and text of the original articles. We also reviewed the entomological literature based on the term *Phlebotomus argentipes* and subheadings *cattle*, *blood meal*, *landing catches* and *breeding*. This search yielded 26 potentially relevant articles, of which 8 were included; 29 additional articles on entomological aspects of the disease were found through citations in the literature and the authors' collections. Other pertinent articles, reports, monographs and book chapters were located through citations or suggested by experts.

## Results

### Risk factors for VL in South Asia

In South Asia, kala-azar classically occurs in agricultural villages where houses are frequently constructed with mud walls and earthen floors, and cattle and other livestock are kept close to human dwellings. Nevertheless, the disease was reported historically from cities such as Calcutta and Madras [21],[22], and poor peri-urban communities in VL-endemic districts may still be vulnerable. The epidemiology of VL is characterized by both large-scale and small-scale spatial clustering. Despite the extensive geographical distribution of *Ph. argentipes* over much of the Indian subcontinent [23] and from Iran to Indonesia [24], VL cases are highly clustered in a small area of northeastern India, southeastern Nepal, and west and central Bangladesh. More than 90% of Indian VL cases occur in the state of Bihar and more than 50% of reported cases in Bangladesh come from 5 subdistricts of Mymensingh district representing less than 2% of the national population [17],[25].

At a much finer spatial scale, living close to a previous case of kala-azar strongly increases risk of both kala-azar and subclinical infection (Table 1) [7], [26]–[28]. In a study of one Bangladeshi community, people living within 50 meters had a 3-fold increase in kala-azar risk and those with a previous case in the same household were 26 times more likely to develop kala-azar, compared to individuals living more than 50 meters away [27]. These findings are thought to reflect the role of kala-azar patients as the most important infection reservoir during the study period. However, new data showing a recent increase in PKDL patients in Bangladesh highlight the likelihood that the role of kala-azar cases as the major leishmanial infection reservoir may not be constant over time [29]. Active case finding was conducted among 22,699 respondents in 2007–2008 in Mymensingh, including the area studied in 2000–2004 [29]. A total of 813 participants (3.6%) reported having had kala-azar since 2002, and 79 (9.7%) of these had developed PKDL by the time of survey, with a median interval of 21 months from kala-azar to onset of PKDL. Eight additional PKDL patients had no antecedent kala-azar history. The annual kala-azar incidence peaked at 85/10,000 in 2004, falling to 46/10,000 in 2007, but PKDL incidence rose steeply from 1/10,000 in 2004 to 21/10,000 in 2007. In Bangladesh, at least, it seems likely that PKDL patients will play an important role in sustaining transmission now and in the near future.

**Table 1:** Risk factors for visceral leishmaniasis disease and infection in South Asia.

Location	Design	Outcome	N	Risk factors	Protective factors	Reference
Bihar, India	Household level case-control	Kala-azar	48 case, 46 control HH	Mud plastered house (4.45, <0.001)^1^, migration into village (3.1, <0.05), vegetation near house (2.8, <0.05), proximity to prior case (not quantified)		[28]
Central Terai, Nepal	Case-control	Kala-azar	84 cases, 105 controls	Cracked mud house walls (2.3, <0.05), palpably damp floor (4.0, <0.01), sleeps outside warm months (2.0, <0.05), laborer as household head (2.8, <0.01)	Owns cow or buffalo (0.34, <0.01), sleeps under bed net in warm months (0.2, p<0.001), sleeps on cot (0.44, p<0.01), ≥3 rooms (0.27, p<0.001)	[30]
Bihar, India	Case-control	Kala-azar	134 cases, 406 controls	Another disease in past year (3.6, <0.01), history of kala-azar in household (1.8, <0.05), mud walls (2.4, <0.001), granary in house (4.3, <0.001), bamboo near house (2.3 (<0.01), house not sprayed in past 6 months (3.4, <0.001)		[31]
Uttar Pradesh, India	Cross-sectional	Kala-azar	2203	Previous kala-azar case in household (42.2, <0.001), sleeps outside, ≥3 people per room, increasing cattle density (1.24, <0.01 in village 1; in village 2), age ≥15 years (2.2, p<0.05)	Bed net use NS^2^ (but ownership low)	[26]
Mymensingh, Bangladesh	Cross-sectional	Kala-azar	2356	Previous kala-azar case in same household (25.6, <0.001) or within 50 m (2.9, <0.001), age 3–45 years (3.7, <0.001)	Always sleeps in net in warm months (0.69, <0.01), each additional cow per 1000 m^2^ (0.81, <0.01)	[27]
Mymensingh, Bangladesh	Hospital-based case-control	Kala-azar	60 cases, 60 controls	Mud house (28.9, <0.001), sleeping on floor (2.1, p not given)	Bed net use NS	[34]
**Studies of subclinical infection**						
West Bengal, India	Cross-sectional	Positive LST	150	Increasing age, proximity to previous case of kala-azar^3^		[10]
Eastern Terai, Nepal	Cross-sectional	Positive serology	373	Proximity to ponds (3.7 [1.6–8.5]^4^), family size ≥6 (4.4 [1.6–12.6]), mud house (3.0 [1.1–7.6]), age ≥15 years (5.5 [1.2–25.0])	Bed net use NS	[32]
West Bengal, India	Retrospective cohort	Seroconversion over 1year	751	Water body within 25 m (2.1 [1.4–4.5]), house dampness (2.4 [1.7–3.7]), livestock ownership (2.1[1.5–3.8]), Muslim religion (1.7 [1.3–2.4])	Sleeps inside (0.6[0.4–0.8]), sleeps clothed (0.5[0.5–0.7]), always sleeps under bed net (0.7[0.5–0.9])	[33]
Mymensingh, Bangladesh	Cross-sectional	Positive LST	1379	Previous kala-azar case in same household (2.86, <0.001) or within 50 m (1.72, <0.01), each 10-year increase in age (1.48, <0.001), additional cow per 1000 m^2^ (1.17, <0.05)	Bed net use NS	[7]
Mymensingh, Bangladesh	Cross-sectional	Positive serology	1379	Previous kala-azar case in same household (1.85, <0.05), each 10-year increase in age (1.12, <0.05)	Bed net use NS	[7]
Mymensingh, Bangladesh	Cross-sectional	Kala-azar vs seropositive	1379	Previous kala-azar case in same household (2.85, <0.01)	Consumption of beef or goat at least twice per month (0.49, <0.05), each 10-year increase in age (0.74, <0.001)	[7]

1 Odds ratio, P value.

2 Not significant.

3 Statistical testing not presented.

4 Odds ratio [95% confidence intervals].

Only six explicit studies of risk factors for kala-azar and four for subclinical infection have been appeared in the international literature since the South Asian VL resurgence began in 1970 (Table 1) [7], [10], [26]–[28], [30]–[34]. The results of these studies should be viewed with caution, because most were cross-sectional studies, which by their nature cannot evaluate the effect of risk factors that could change over time. In the case of case-control studies, use of an appropriate control group is also essential to yielding valid assessments of risk. Nevertheless, a number of fairly consistent themes emerge, that can help inform thinking about interventions to control the disease. In the majority of studies, sleeping on the ground or outside was associated with increased risk of kala-azar, whereas sleeping on a cot was protective. Bed net use was protective against kala-azar in two studies [27],[30] but showed no significant effect in two others [26],[34]. Net condition, utilization prevalence, and effectiveness of insecticide impregnation, where applicable, may all affect the degree of protection associated with nets. Net use showed no protective effect against subclinical infection in 3 of 4 analyses [7],[32],[33]. In general, kala-azar risk was higher for children and young adults than for older adults who have a high prevalence of protective DTH responses [9]. However, in new foci where exposure is relatively recent, older adults may have an equally high risk of kala-azar [26]. Infants and young toddlers were relatively spared, perhaps due to being protected under a blanket or bed net during the hours of peak sand fly activity and consequently having less cumulative exposure [27].

Important environmental risk factors include living in a house with mud-plastered walls, especially if the walls are cracked; damp earthen floors; and closeness to small bodies of water and vegetation [30]–[33]. These factors are hypothesized to facilitate sand fly survival and increase vector abundance by providing breeding sites, diurnal resting places and humidity. The typical housing characteristics, as well as low educational level, lack of land, and other socioeconomic indicators, highlight the increased risk associated with poverty [26],[30],[35],[36]. Poverty mediates increased VL risk via multiple pathways, including increased sand fly access into poorly constructed houses and increased human exposure due to lack of personal protective measures such as nets [36]. However, within communities where characteristics of the local microenvironment are disproportionately influential, relative poverty may not be identified as a significant risk factor [27],[37].

Household members share more than just common exposures to potentially infected sand flies; genetic and nutritional factors may also be operative in this setting. In Brazil, specific gene loci coding for aspects of the immune response have been found to be associated with increased risk of symptomatic zoonotic VL in humans and dogs (the reservoir) [38]–[41], and poor nutritional status has been shown to increase the risk of progression from infection to kala-azar [42],[43]. Although similar data are not yet available for South Asian VL, both genetic and nutritional factors are assumed to play a role [7],[39]. Community-based data from Bangladesh demonstrated that population-level micronutrient status was poor, and higher (though still meager) intake of beef or goat increased the likelihood that an infection would remain asymptomatic rather than progressing to kala-azar [7]. Population status with respect to zinc, retinol, and other micronutrients known to modulate immunological status may be important in determining the proportion of infected individuals who become ill [7]. One study reported that non-leishmanial illness in the previous year increased kala-azar risk [31]; this factor may also act through a detrimental effect on immune status.

### The role of cattle in sand fly ecology and the VL transmission cycle

Data for the effect of cattle and other large livestock on human risk are contradictory, possibly reflecting the diversity of study designs and differences in statistical rigor. Ownership or proximity of livestock was associated with significant protection in some studies [27],[30], whereas, in others, kala-azar risk appeared to increase for those living in close proximity to cattle [26]. In Nepal, ownership of a cow or buffalo was associated with a significant decrease in kala-azar risk, but because of the case-control design, the authors were unable to conclude that protection was due to the entomological effects of cattle, as opposed to large livestock acting as an indicator of higher socio-economic status [30]. In the Bangladesh community-based study, cattle ownership was not associated with statistically significant protection, but each additional cow per 1000 m^2^ around an individual's house decreased the risk of kala-azar by approximately 20% [27]. In other words, people were protected from kala-azar by their neighbors' cattle as well as their own. In the same study, the likelihood of a protective DTH response increased with increasing cattle density [7]. Interestingly, Napier and Das Gupta noted in 1931 that householders that kept cattle close to their dwelling places had a lower incidence of kala-azar [44]. A similar apparent contradiction is seen in African VL studies: a village-based study in eastern Sudan showed increased risk associated with cattle ownership for the first 3 years of a 5-year study [45], whereas a case-control study in Uganda indicated that treating livestock with topical insecticide against ectoparasites increased kala-azar risk, suggesting displacement of sand fly feeding to humans [46].

The complexity of the effect of cattle on VL risk may spring from the multiple ways in which their presence can alter sand fly density, infection rates and human-sand fly exposure, and to the complex and not yet fully elucidated population dynamics and behavior of *Ph. argentipes* (Table 2). Like the New World VL vector *Lutzomyia longipalpis*, *Ph. argentipes* is a predominantly peridomestic sand fly. *Ph. argentipes* breeds in moist organic soils at the junction of the floor and walls of cattle sheds and earthen houses [47]–[51]. Recent entomological trials of long-lasting insecticide-treated nets (LLINs) in Bihar failed to reduce indoor female *Ph. argentipes* densities, leading the authors to conclude that breeding may occur predominantly outside houses [52]. *Ph. argentipes* shows opportunistic feeding habits similar to those of *Lu. longipalpis*, whereby host “preference” is likely to be determined by host accessibility, abundance, size and biomass [53]. *Ph. argentipes* blood meals comprise predominantly bovine and human blood, influenced by trap positioning and trapping efficiency [54]. Although *Ph. argentipes* can be collected on bovine or human bait, several-fold more flies are generally caught on cattle than humans over the same time period [55] which probably reflects the influence of host density or biomass on sand fly feeding behaviour. As expected, blood meals in *Ph. argentipes* captured in cattle sheds and human dwellings reflect the predominant host (Table 2). Where humans and cattle are housed under the same roof, 19% and 66% of bloodmeals are of human and bovine origin, respectively [56]. In cattle sheds, 81–92% are bovine and 1–22% are human blood, and in human dwellings 49–100% are human and 0–39% are cattle blood [56]–[59], reflective of the complex dynamics of vector-host contact rates and consequences for transmission.

**Table 2:** *Phlebotomous argentipes* ecology, cattle and human risk of leishmaniasis.

Characteristic	Evidence	References
Presence of cattle decreases leishmanial risk in some studies, increases risk in others	Decreased risk of kala-azar with ownership of cattle in Nepal	Bern, 2000 #232
	Decreased risk of kala-azar with increased cattle density in Bangladesh	Bern, 2005 #352
	Increased risk of kala-azar with increased cattle density in India	Barnett, 2005 #390
	Increased risk of positive leishmanin skin test with increased cattle density in Bangladesh	Bern, 2007 #755
	Increased risk of seroconversion with livestock ownership in India	Saha, 2008 #855
*Phlebotomous argentipes* seasonality and activity	Peak abundance in summer, smaller peak after rainy season	[51],[55],[73],[93]
	Active dusk to dawn, peak activity 23:00–24:00	
	Human xenodiagnosis studies – <1% positive during day versus 5% at night	
Cattle provide mating aggregation site for *Ph. argentipes*, male pheromone recruitment likely	Male: female sand fly ratios high early, decrease later at night	[55],[60],[61]
	*Ph. argentipes* swarms on cattle with spacing and wing flapping similar to that found in mating aggregations of *Lu longipalpis*	
*Ph. argentipes* feed opportunistically from cattle and humans	Blood meals in cow sheds: median 84.4% bovine/14.9% human	[55]–[60]
	Blood meals in human dwellings: 21.7% bovine/69.7% human	
	Landing/biting catches higher on cattle than humans	
*Ph. argentipes* breeding sites in cow shed and surroundings	Larvae found in cow shed and human dwelling usually at base of walls	[47]–[51]
	Highest number of immature forms inside cow shed and in loose soil outside cow shed	
Feeding on cattle may decrease infection rate in sand flies	Multiple negative studies seeking leishmanial infection in animals	[94]–[96]

In Bihar, peak *Ph. argentipes* abundance has been documented in March-June (summer) and in October (just after the rainy season) and peak biting activity is seen late in the evening [55]. *Ph. argentipes* seasonality is similar in Bangladesh (unpublished data, Chowdhury, Dotson and Bern). Reports of *Ph. argentipes* “swarming” on cattle [60],[61], non-random spacing [60] and changing male to female ratios with time of night [55],[61] strongly suggest that *Ph. argentipes* forms leks for mating and blood feeding. This behavior and its implications for control interventions have been more fully described for the New World peridomestic sand fly *Lu. longipalpis* [54],[62]. Attracted by host kairomones, *Lu. longipalpis* males colonize the backs of animal hosts in the early evening, releasing long-range aggregation pheromones which attract more male and female flies [54],[62],[63]. For *Lu. longipalpis*, female recruitment reaches carrying capacity before that for males, such that the equilibrium male to female ratio is high [62]. Female blood feeding success (which relates to survival and fecundity) diminishes with increasing female fly aggregation density, which may then increase emigration to alternative aggregation sites [63]. Disrupting male recruitment pheromone production by insecticide application may lead to increased attraction to unsprayed sites or unprotected hosts. For example, spraying of animal sheds in Brazil resulted in increased sand fly densities in untreated dining huts [62], indicating that incomplete coverage of all aggregation sites could lead to increased sand fly abundance in vulnerable human dwellings and increased leishmanial transmission to humans. In summary, the impact of cattle on human kala-azar risk represents the outcome of a complex combination of effects on sand fly abundance, access and attraction to blood meal sources, and sand fly infection rates. Interventions that alter sand fly access to cattle should be carefully evaluated with respect to their impact on human exposure.

## Discussion

### Lessons for the elimination program and key unanswered questions

**Figure 1:**
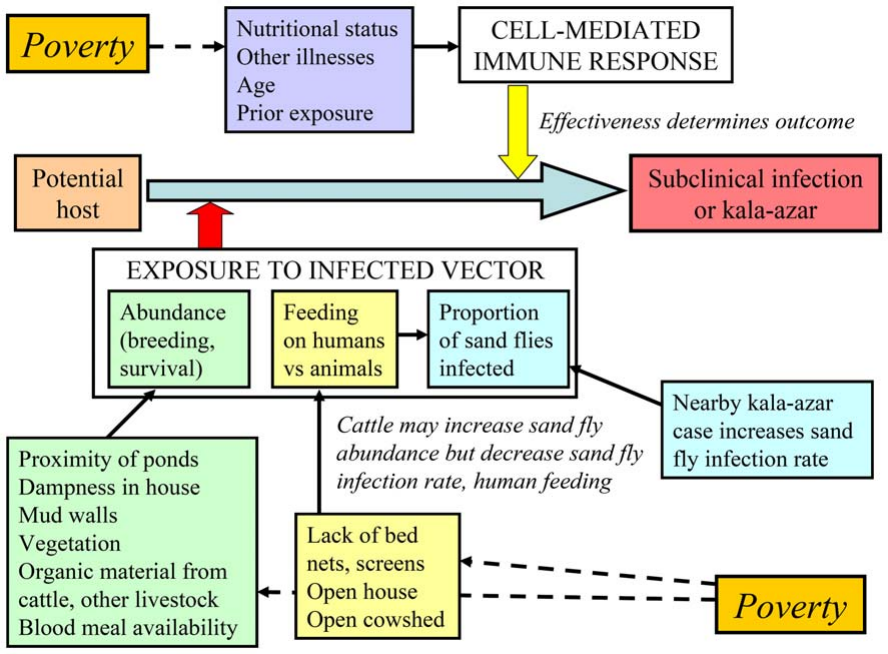
Diagram of the interplay of factors that affect risk of kala-azar and asymptomatic *Leishmania donovani* infection in South Asia. Kala-azar risk is increased by proximity to the infectious reservoir host (an untreated kala-azar patient) and by exposure to infected sand flies, but may be decreased by behaviors such as bed net use that interrupt human-sand fly contact or host factors such as diet that affect the immune response to the parasite. Cattle may affect risk in complex ways, through their effect on sand fly abundance, breeding, infections rates and feeding frequency on humans.

The studies reviewed in this article demonstrate that patterns of VL occurrence in South Asia are determined by the interplay of factors affecting sand fly abundance, infection rates and feeding behavior; proximity of infectious persons, especially kala-azar patients; population- and individual-level susceptibility; and determinants of human exposure to infected sand flies (Figure 1). This web of interactions offers opportunities for and lessons regarding control strategies (Boxes 1 and 2), and highlights fundamental gaps in the evidence base needed to promote the success of the current VL elimination initiative (Box 3).

**Box 1. Implications for the Human Side of the Control Effort**

- Early kala-azar diagnosis and effective treatment are essential to decrease infection reservoir.
- PKDL poses grave threat to control effort, which is compounded by the current difficulty of treatment.
- Even if kala-azar elimination is achieved, many subclinically infected people will remain.
  - Need to assess importance as an infection reservoir
  - Economic development and nutritional improvement may help to decrease risk that this potential reservoir will fuel later resurgence
- HIV-VL coinfection will complicate treatment and provide a more infectious group of patients, and needs to be addressed through.
  - surveillance for coinfection
  - efforts to ensure prompt entry of coinfected patients into comprehensive care
  - aggressive vector control

**Box 2. Implications for Vector Control Programs**

- Must achieve and maintain good vector control with broad coverage of communities around those with reported high kala-azar rates.
- Must maintain for years because of PKDL.
- Housing improvement and environmental interventions may be useful adjunct measures.

**Box 3. Crucial Questions That Need to be Addressed through Carefully Designed Demonstration Projects**

- Can VL be eliminated from an area?
- What would it take to prevent its rapid resurgence (as happened in the 1970s)?
- How can we institute locally appropriate, sustainable VL surveillance systems to monitor progress and document elimination?
- What is the best combination and sequence of vector control measures for a given site, and what are the logistics for implementing these?
- How long do the insecticide-treated nets proposed for use in current control programs maintain their entomological effectiveness under field conditions and how can they be optimized?
- Are asymptomatically infected individuals infectious to sand flies and do they constitute an epidemiologically important infection reservoir?
  - How does their level of infectiousness compare to that of kala-azar and PKDL patients?
  - If some asymptomatically infected individuals are infectious and others not, are there markers that can distinguish those who are infectious?
- What are the best methods for surveillance and monitoring in each major site?
- How can we develop better active case finding methods, diagnostic tests and treatment regimens for PKDL?

The spatial patterns, especially at the community level, underscore the role of kala-azar patients as the major infection reservoir during periods of high disease incidence. Minimizing their infectious period through “rapid diagnosis and highly effective treatment” is appropriately a high priority within the South Asian control strategy, and the availability of new drugs and combination regimens has greatly improved the prospects for safe, successful kala-azar therapy [64]–[67]. However, PKDL treatment continues to rely primarily on courses of 120 intramuscular injections of sodium stibogluconate [68]. PKDL is less likely than kala-azar to prompt patients to seek care, because of the lack of systemic illness, and is difficult to treat effectively because of the prolonged drug course. If, as seems likely from xenodiagnosis studies, PKDL patients provide an important infection reservoir, the rising incidence of PKDL documented in Bangladesh poses a grave threat to VL elimination [13],[14],[29].

The relative importance of kala-azar, PKDL and asymptomatic infections as reservoirs in the transmission cycle is currently unknown. In one study in India, *Leishmania* parasites were visualized in peripheral blood smears of persons with asymptomatic infection [69]. In a Brazilian study, 25% of sand flies fed on kala-azar patients but none of those fed on asymptomatically infected humans became infected [70]. In Spain, HIV-*L. infantum*-co-infected patients were shown to be highly infectious to sand flies [71]; an increase in co-infection rates in South Asia would increase the effective infection reservoir. Based on sparse data from selected Indian patients, the proportion (8/8 PKDL, 15/18 kala-azar patients) that infected laboratory-reared *Ph. argentipes* and the percentage of sand flies infected (15–53%; 15–42%) were roughly similar for PKDL and kala-azar [13], [14], [72]–[77] (reviewed in [4]), but to date there have been no unbiased population studies of xenodiagnosis in anthroponotic VL.

Xenodiagnosis studies of dog populations are more numerous, and demonstrate that asymptomatic and presymptomatic *L. infantum*-infected dogs infect sand flies, but generally with lower frequency than dogs with symptomatic disease [78],[79]. Nevertheless, in some studies, a majority of asymptomatically infected dogs were infectious to sand flies [80],[81]. A recent meta-analysis demonstrates that the average percentage of dogs infectious to sand flies increased significantly with increasing clinical severity, from 29% of asymptomatic dogs to 80% of polysymptomatic dogs [4]. These findings, together with human epidemiological data, suggest that at least some asymptomatically infected humans may have the potential to transmit leishmaniasis, but are likely to be much less infectious than patients with active kala-azar or PKDL. However, at least 125 million people live in VL-endemic areas in South Asia, and perhaps 10 to 30% of these people have asymptomatic leishmanial infection. Even limited infectiousness of a fraction of these people could represent a large reservoir of infection and jeopardize efforts to eliminate the disease.

The failure of targeted vector control activities in South Asia since the 1990s suggests that vector programs for VL elimination need to be proactive, rather than reactive, and cover broad geographic areas. Current vector control strategies rely on IRS targeted to villages or parts of villages, in response to reported incidence of kala-azar in national passive surveillance systems [64]. Indeed, data from Bangladesh suggest that the delay between case detection and reactive IRS was so lengthy that it had no impact on transmission [27],[29]. The success of insecticide-treated nets against cutaneous leishmaniasis and sand fly vectors is well documented [82]–[88]. We hypothesize that VL control in South Asia could be more effective if IRS with broad coverage over VL-endemic areas is used to rapidly decrease sand fly populations and parasite transmission, and is simultaneously combined with active case finding and treatment, and widespread distribution of insecticide-treated nets to prevent the return of transmission from remaining kala-azar and PKDL patients when sand fly populations recover. Insecticide-treated nets could help fill the gaps that often result from the logistical difficulties of a sustained IRS program. Surveillance for HIV-VL coinfection and mechanisms to ensure that HIV-coinfected patients have access not just to rapid diagnosis and treatment of VL, but also antiretroviral therapy, secondary prophylaxis and insecticide-treated nets, will be necessary to ensure good clinical response and avoid an increase in the effective leishmanial infection reservoir. Human dwellings in South Asia are vulnerable to sand fly entry, even if breeding and aggregation occur predominantly in animal sheds. The effects of insecticide treatment of cattle sheds need to be evaluated, paying particular attention to the possibility that insecticide could repel sand flies resulting in higher biting rates on nearby unprotected humans. A simple intervention based on application of lime to mud walls showed promising entomological effects in a small pilot study [89]; further study to evaluate effectiveness and safety could add another inexpensive vector control method. Mitigation of the damp earthen floors and cracked mud walls of rural houses, and installation of screens or curtains to decrease sand fly access, could also play a role in decreasing human exposure to potentially infected sand flies, similar to housing improvement programs for control of Chagas disease [90].

Finally, crucial gaps remain in our knowledge base to achieve VL control (Box 3). The regional elimination program relies on the transient near-elimination of kala-azar during the malaria eradication era as a surrogate for proof of principle, while ignoring the rapid, explosive resurgence that occurred afterwards. Moreover, recent data are inadequate as a basis to design a control strategy with a high likelihood of lasting impact on VL incidence. Although a number of studies of the impact of insecticide-treated nets have been or are being conducted [91],[92], the resulting data may not provide a sufficient basis for elimination program policy decisions, given the complexity of the topic. As seen in the current review, the epidemiological determinants of kala-azar and subclinical infection are not necessarily the same; the use of seroconversion as the major outcome in an intervention trial may lead to erroneous conclusions about risk for disease. Similarly, sand fly density by itself does not predict infection risk; for example, successful CL control has been achieved in the absence of any measureable impact on the vector population [85]. In addition, no data are currently available to assess the duration of effectiveness of long-lasting impregnated nets under the conditions of usage in South Asian villages. To ensure the success of the initiative, it is more urgent than ever to conduct adequately powered, integrated demonstration projects to provide direct proof of principle for VL elimination. Several demonstration projects in carefully chosen sites may be necessary to ensure that the ultimate strategies are appropriate to the ecological and epidemiological characteristics of each location.

After kala-azar incidence is below the program threshold of 1/10,000 population, regional program documents propose a maintenance phase of 2–3 years [15],[64]. At the end of this period, sand fly populations are likely to rebound rapidly, as they did in the 1970s [16]–[20]. Based on the experience of the 1970s, maintenance activities will be necessary for a prolonged period of time, but currently there are no data to plan this critical phase. It is important to determine the most effective combination of maintenance activities and their necessary duration before national programs must make decisions on maintenance strategies and certification requirements. Finally, the resurgence in the 1970s was widely believed to have been sparked by PKDL patients who remained untreated for years [13], but the standard PKDL treatment regimen still requires 120 injections of a toxic drug. More effective, short-course treatment regimens for PKDL are urgently needed.

The South Asian regional initiative was begun with the admirable goal of eliminating the crushing burden of a lethal disease in some of the poorest populations of the world. Unfortunately, the evidence base to ensure the success of the initiative is still lacking. Without a coordinated, scientifically rigorous effort to fill these gaps in the near future, the current unparalleled opportunity to make a sustained impact on the disease burden caused by VL in South Asia may be lost.

## Supporting Information

Checklist S1 PRISMA checklist.
(0.07 MB DOC)

Figure S1 PRISMA flowchart.
(0.06 MB DOC)
