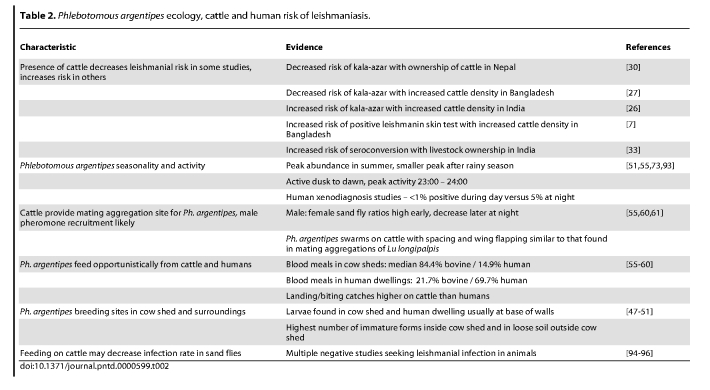# Correction: Of Cattle, Sand Flies and Men: A Systematic Review of Risk Factor Analyses for South Asian Visceral Leishmaniasis and Implications for Elimination

**DOI:** 10.1371/annotation/f96dd2ce-05de-4aa5-a9d5-c481b0745c84

**Published:** 2010-03-05

**Authors:** Caryn Bern, Orin Courtenay, Jorge Alvar

In the first five rows of the References column, the numbered citations were incorrect. The corrected Table 2 can be found at 

**Figure:**