# Supplementary material for: Of Cattle, Sand Flies and Men: A Systematic Review of Risk Factor Analyses for South Asian Visceral Leishmaniasis and Implications for Elimination
Source: PLoS Negl Trop Dis. 2010 Feb 9;4(2):e599. doi: 10.1371/journal.pntd.0000599 (PMC2817719; doi:10.1371/journal.pntd.0000599)
Supplement: Figure S1 — PRISMA flowchart. (0.06 MB DOC) [file pntd.0000599.s002.doc]

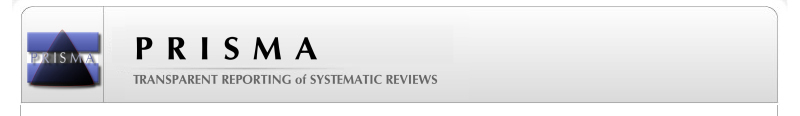
**PRISMA 2009 Flow Diagram**

**Screening**

**Included**

**Eligibility**

**Identification**

Records identified through database searching
(n = 26 )

Additional records identified through other sources
(n = 0)

Records after duplicates and review articles removed
(n = 10 )

Records screened
(n = 10 )

Records excluded
(n = 0 )

Full-text articles assessed for eligibility
(n = 10 )

Full-text articles excluded, with reasons
(n = 0 )

Studies included in qualitative synthesis
(n = 10 )

Studies included in quantitative synthesis (meta-analysis)
(n = NA )
